# Activation of LncRNA FOXD2‐AS1 by H3K27 acetylation regulates VEGF‐A expression by sponging miR‐205‐5p in recurrent pterygium

**DOI:** 10.1111/jcmm.16024

**Published:** 2020-10-23

**Authors:** Yali Gao, Xiaoling Luo, Jun Zhang

**Affiliations:** ^1^ Department of Ophthalmology Shenzhen People’s Hospital (The Second Clinical Medical College, Jinan University) Shenzhen China; ^2^ Department of Obstetrics and Gynecology Shenzhen People’s Hospital (The Second Clinical Medical College, Jinan University) Shenzhen China

**Keywords:** FOXD2‐AS1, H3K27 acetylation, long noncoding RNA, miR‐205, Pterygium, VEGF‐A

## Abstract

LncRNA FOXD2‐AS1 is abnormally expressed in many diseases. However, the molecular mechanisms whereby FOXD2‐AS1 is involved in recurrent pterygium remain unknown. Here, qRT‐PCR was performed to quantify FOXD2‐AS1 expression, while CCK‐8, flow cytometer and neoplasm xenograft assays were used to investigate its function. Dual‐luciferase reporter, RIP and RNA pull‐down assays were conducted to address the relationship between FOXD2‐AS1, miR‐205‐5p and VEGF‐A, while ChIP assays were used to detect H3K27 acetylation at the FOXD2‐AS1 promoter. FOXD2‐AS1 expression was up‐regulated in recurrent pterygium tissues. Moreover, a high FOXD2‐AS1 expression was associated with advanced stages, increased microvessel density and shorter recurrent‐free survival. In addition, ROC analysis showed that FOXD2‐AS1 is a valid predictor of recurrent pterygium. Furthermore, we show that FOXD2‐AS1 induced proliferation and inhibited apoptosis in a cell line derived from recurrent pterygia (HPF‐R) at least partially through the regulation of the miR‐205‐VEGF pathway. In addition, the up‐regulation of FOXD2‐AS1 was attributed to the H3K27 acetylation at the promoter region. In conclusion, FOXD2‐AS1 is activated via its H3K27 acetylation and regulates VEGF‐A expression by sponging miR‐205‐5p in recurrent pterygium. Our results may provide a basis for the development of new therapeutic targets and biomarkers for recurrent pterygium.

## 1 INTRODUCTION

Pterygium, a common ocular surface disorder, is an elastotic degeneration of conjunctival tissue with abnormal growth of fibroblasts and blood vessels invading the cornea across the limbus, which can lead to impaired vision. Pterygium may mimic tumorigenesis and is thought to be the result of altered epithelial cell proliferation and angiogenesis.^1^ The most challenging aspect of pterygium treatment is the high incidence of recurrence (from 1% to 30%).^2^ Conjunctival autograft is the most common procedure to avoid recurrence. However, it can be associated with several complications, including inflammation, infections, button holes, suture abscesses, prolonged operative time and postoperative discomfort.^2^ Therefore, finding target sites for treatment and novel biomarkers for recurrence have constituted the major goals in pterygium research.

Long noncoding RNAs (lncRNAs) are defined as non‐protein‐coding RNAs longer than 200 nucleotides. Several studies have associated lncRNAs to biological processes in human cancers, such as cell proliferation, apoptosis, invasion and angiogenesis.^3^, ^4^, ^5^ In recent years, studies on the role of lncRNAs in pterygium have also made some progress. Liu et al identified 3066 up‐regulated and 1646 down‐regulated lncRNAs in pterygium tissues compared with paired adjacent normal conjunctival tissues.^6^ Lan et al reported that linc‐9432 is up‐regulated in pterygium and regulates the levels of differentiation‐related transcripts in fibroblasts.^7^ In addition, lncRNAs have also been described as candidate biomarkers for prognosis. For instance, lncRNA‐MEG3 is a proven biomarker for retinoblastoma and cervical cancer.^8^, ^9^ Therefore, noncoding RNAs are a new promising source of disease biomarkers that can be applied in clinical practice.

Long noncoding RNA FOXD2 adjacent opposite strand RNA 1 (lncRNA FOXD2‐AS1, NR_026878) is located on chromosome 1p33 and has a transcript length of 2527 nucleotides. Overexpression of FOXD2‐AS1 causes malignant cell proliferation and the down‐regulation of several tumour suppressor genes.^10^, ^11^ However, the expression level and function of FOXD2‐AS1 in recurrent pterygium have not been reported yet. In addition, it remains unclear whether FOXD2‐AS1 levels can be used as a biomarker for recurrent pterygium. Thus, we sought to answer these questions.

In this study, we found overexpression of FOXD2‐AS1 in recurrent pterygium tissues and that the expression levels were associated with disease stage, microvessel density (MVD) and recurrent‐free survival. Moreover, FOXD2‐AS1 had predictive value in recurrent pterygium. In addition, function assays revealed that FOXD2‐AS1 induces cell proliferation and inhibits cell apoptosis both in vitro and vivo. Furthermore, our results suggest that FOXD2‐AS1 may act as a competing endogenous RNA (ceRNA) to regulate the miR‐205‐VEGF pathway in HPF‐R cells and that H3K27 acetylation activates FOXD2‐AS1 expression. In conclusion, FOXD2‐AS1 promotes pterygium growth, at least partially, through the regulation of the miR‐205‐VEGF pathway. Thus, we propose FOXD2‐AS1 as a potential biomarker for recurrent pterygium.

## 2 MATERIALS AND METHODS

### 2.1 Tissue samples

Surgical specimens from 126 pterygium tissues (primary = 104, recurrent = 22) and matched adjacent conjunctiva tissues were obtained between April 2017 and June 2018 from patients who underwent surgery at the Shenzhen People's Hospital. The diagnosis and histological grading were approved by pathologists. The study was approved by the ethics committee of the Shenzhen People's Hospital, and written informed consent was obtained from each participant according to the Declaration of Helsinki. The patients’ information is summarized in Table 1.

**Table 1:** Clinical features of the patients

Clinical variables	Primary pterygium (n = 104)	Recurrent pterygium (n = 22)	*P* value
Age (mean ± SD)	49.8 ± 13.1	48.5 ± 11.7	.635
MVD (mean ± SD)	15.2 ± 3.3	18.7 ± 3.8	<.001
Stage			
Inflamed	66 (63.5%)	19 (86.4%)	.045
Quiescent	38 (36.5%)	3 (13.6%)	
Gender			
Male	48 (46.2%)	9 (40.9%)	.814
Female	56 (53.8%)	13 (59.1%)	

Abbreviation: MVD, Microvessel density.

### 2.2 Cell lines established from of recurrent pterygia (HPF‐R)

Freshly collected recurrent pterygium tissues were cut into small sections, washed in Hanks solution, and incubated in DMEM medium (Invitrogen, Carlsbad, California, US) with 100 mmol/L sorbitol (Sigma, Missouri, US) and 50 μg/mL dispase II (Invitrogen) for 60 minutes at 37°C. Then, pterygium cells were dissociated using TryPLE reagent (Invitrogen) for 5 minutes at 37°C. The isolated cells were cultured in DMEM medium supplemented with 10% FBS and gentamicin (50 g/mL) at 37°C in a humidified atmosphere with 5% CO_2_.

Purification of cell: First, we used flow cytometry to separate p63 (+) and pan cytokeratin (+) pterygium cells.^12^, ^13^, ^14^ Next, we observe the cell morphology under an inverted phase‐contrast microscope (Olympus, Tokyo, Japan) and scrape off other cells using cell scraper. Stable cells passaged for 3 to 7 generations were used in the experiments. C646 (Selleck Chemicals, Houston, TX, USA) was used at 10 μmol/L for 48 hours as required.

### 2.3 Establishment of stable cell lines

Lentiviruses carrying FOXD2‐AS1 plasmids or expressing shRNA against FOXD2‐AS1 were constructed by Genechem (Shanghai, China). Transfection of HPF‐R cells with lentiviral vectors was performed with Lipofectamine 3000 (Invitrogen) according to the manufacturer's instructions. Puromycin (2 μg/mL)‐resistant clones were picked and separated, and green fluorescent protein signals were checked using fluorescence‐activated cell sorting. The shRNA sequences used are listed in Additional file 1: Table S1.

### 2.4 RNA isolation and quantitative real‐time PCR

Total RNA from tissues and cells was purified using Trizol reagent (TaKaRa, Otsu, Japan). One Step PrimeScript Kit (TaKaRa) was used for the reverse transcription of isolated RNA. Moreover, qRT‐PCR was carried out using a SYBR Premix Ex Taq kit (TaKaRa) and the CFX96 Real‐Time PCR Detection System (Bio‐Rad, Hercules, California, USA). *GAPDH* was used as a normalization control, and the relative expression level was calculated using the 2^−ΔΔCt^ method. FOXD2‐AS1 primers were purchased from Generay Biotech (Shanghai, China) and the sequences are listed in Additional file 2: Table S2.

### 2.5 Western blot

Cells were lysed with RIPA lysis buffer supplemented with a protease inhibitor cocktail. Then, total protein extractions (50 μg) were separated using 10% SDS‐polyacrylamide gel electrophoresis and transferred onto PVDF membranes. After blocking in TBST with 5% skimmed‐milk for 1 hour, the membranes were incubated with VEGF‐A primary antibody (1:1000; Cell Signaling Technology) at 4°C overnight, followed by incubation with HRP Goat‐anti‐Rabbit (1:2000; Santa Cruz Biotechnology) at room temperature for 2 hours. Bound proteins were visualized using an enhanced chemiluminescence system (ECL, Thermo Scientific) and GAPDH (1:1000; Cell Signaling Technology) was used as an internal control. The quantification of Western blot signals was performed by densitometry with G:BOX XT4 (Syngene, Cambridge, UK).

### 2.6 Immunofluorescent staining

Cells were washed with cold‐PBS three times, fixed with 4% formaldehyde for 60 minutes and permeabilized using 0.1% triton‐x for 30 minutes. After blocking with 10% normal goat serum for 1 hour at room temperature, the samples were incubated with primary antibodies (anti‐VEGF‐A; 1:1000; Cell Signaling Technology, Massachusetts, USA) for 1 hour at room temperature and gently washed three times in PBS. Then, the samples were incubated with fluorescein (FITC)‐conjugated secondary antibody (1:1000; Cell Signaling Technology) and stained with DAPI (1 μg/mL, Cell Signaling Technology) before image acquisition using a fluorescence microscope (Olympus, Tokyo, Japan).

### 2.7 Cell proliferation assay

Cell proliferation was measured using a Cell Counting Kit‐8 (CCK‐8) assay (Dojindo, Kumamoto, Japan). Transfected HPF‐R cells were seeded in 96‐well plates at a density of 10 × 10^3^ cells per well. CCK‐8 (10 μL) was added to each well at the indicated time points, and plates were incubated for 3 hours at 37°C. Then, the absorbance at 450 nm was measured with Multifunctional microplate reader SpectraMax M5 (Molecular Devise, California, USA).

### 2.8 Cell apoptosis assay

Cell apoptosis was detected using the Annexin V‐FITC Apoptosis Detection Kit (eBioscience, San Diego, CA, USA) and a BD FACSCalibur flow cytometer (BD Biosciences, California, USA), according to the manufacturer's instructions. The level of apoptosis was assessed using Cell Quest software (BD Biosciences) and the relative ratio of early apoptotic cells was calculated for further comparisons.

### 2.9 Neoplasm xenograft assay

Twelve BALB/c nude mice (6‐8 weeks old) were randomly and equally divided into four groups (Three mice per group). Approximately 1 × 10^7^ HPF‐R1 cells (FOXD2‐AS1 overexpression), HPF‐R2 cells (FOXD2‐AS1 knockdown) or their controls were subcutaneously injected into mice. The length and width of the tumours were measured with a Vernier caliper every week, and the tumour volume was calculated using the following formula: volume = (length × width^2^)/2. After 4 weeks, all mice were sacrificed and xenograft neoplasms were weighed. Protocols for animal studies were approved by the ethics committee of the Shenzhen People's Hospital.

### 2.10 Dual‐luciferase reporter assay

Cells were cultured in 12‐well plates and co‐transfected with luciferase reporter constructs of target genes (wild type or mutated type 3’‐UTRs respectively, Genearray Biotechnology, China) and miR‐205‐5p mimics using Lipofectamine 3000 (Invitrogen). After 48 hours, luciferase activity was measured using the Dual‐Luciferase reporter assay system according to the manufacturer's instructions (Promega, Fitchburg, WI, USA). Relative luciferase activity was normalized to Renilla luciferase activity.

### 2.11 RNA immunoprecipitation (RIP)

RIP™ RNA‐Binding Protein Immunoprecipitation Kit (Millipore, Billerica, MA, USA) was used for RIP according to the manufacturer's protocol. Briefly, cells were lysed using complete RIP lysis buffer and incubated with magnetic beads conjugated with anti‐argonaute‐2 antibody (AGO2, (1:1000; Abcam, San Francisco, USA) or control anti‐IgG antibody for 6 hours at 4°C. After protein removal from the beads, RT‐qPCR analysis of the purified RNA was conducted to verify the presence of target RNA.

Biotin‐labelled miR‐205‐5p‐WT, miR‐205‐5p‐MUT and control probe were purchased from Geneseed Biotech (Shanghai, China). Cells were lysed with lysis buffer and incubated with specific probes. Then, the cell lysates were incubated with M‐280 streptavidin magnetic beads (Invitrogen, San Diego, CA, USA) to pull‐down the biotin‐labelled RNA complex according to the manufacturer's protocol. The bound RNAs were purified using Trizol for qRT‐PCR analysis.

### 2.12 Chromatin immunoprecipitation assay (ChIP)

ChIP was conducted using EZ ChIP™ Chromatin Immunoprecipitation Kit (Millipore, Bedford, MA, USA) following the manufacturer's instructions. Formaldehyde was applied to generate DNA‐protein crosslinks for 20‐30 minutes. After that, the lysate was sonicated to reduce the DNA length to 200‐500 bp. The chromatin extract was incubated with magnetic beads and either anti‐H3K27ac (1:1,000; Abcam, Cambridge, UK) or anti‐IgG (negative control) antibody. Then, the immune complex was eluted, the protein was unlinked, and the associated DNA fragments were purified. Precipitated DNA was analysed using qRT‐PCR. The ChIP data were calculated as the percentage relative to the input DNA using the equation 2 [Input Ct − Target Ct] ×0.1 × 100. The primers for the FOXD2‐AS1 promoter are listed in Additional file 2: Table S2.

### 2.13 Statistical analysis

SPSS software package 13.0 was used to perform statistical analyses, and the results are shown as the mean ± SD from three separate experiments. Data were analysed using two‐tailed Student's t test and χ^2^ test, Fisher's exact test or Wilcoxon test, as appropriate. Pearson correlation analysis was performed to investigate correlations. A receiver operating characteristic (ROC) curve was established to evaluate the diagnostic value for recurrence prediction. The odds ratio (OR) was calculated using logistic regression analysis, and Hazard Ratio (HR) was calculated using Cox regression analysis. Recurrent‐free survival rates were calculated using the Kaplan‐Meier method with the log‐rank test applied for comparison. The statistical significance level was set at *P* < 0.05. All experiments were conducted in triplicates.

## 3 RESULTS

### 3.1 Correlation between FOXD2‐AS1 and clinicopathologic characteristics of pterygium patients

**Figure 1:**
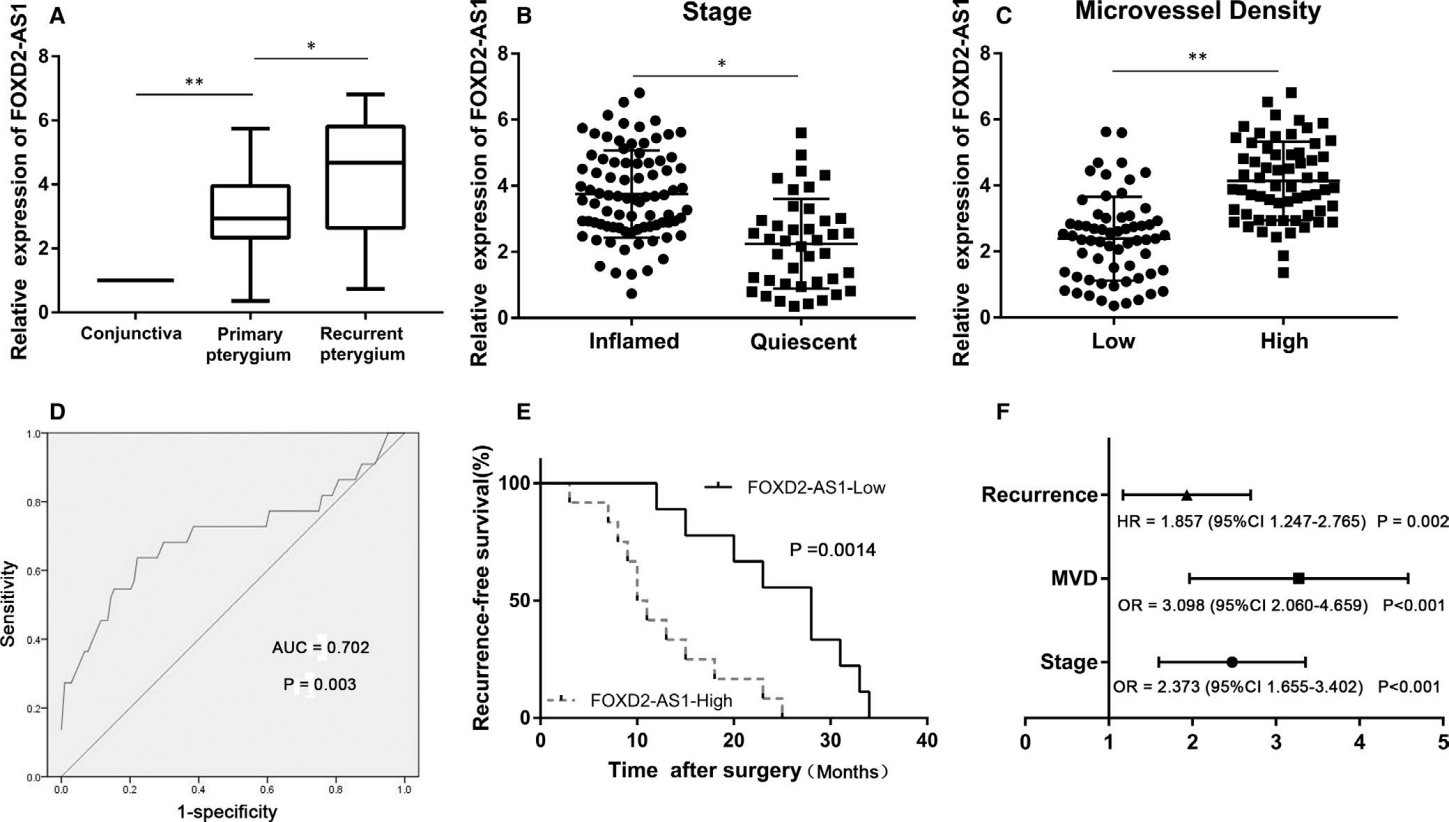
FOXD2‐AS1 expression and its clinical significance in pterygium. A, Relative expression of FOXD2‐AS1 in matched adjacent conjunctiva tissues (n = 126), primary pterygium tissues (n = 104) and recurrent pterygium tissues (n = 22). B, Relative expression of FOXD2‐AS1 at different stages of pterygium. C, Relative expression of FOXD2‐AS1 in MVD‐low group (n = 63) and MVD‐high group (n = 63). D, Evaluation of the clinical value of FOXD2‐AS1 for recurrence of pterygium using the ROC curve. E, Kaplan‐Meier analysis for recurrent‐free survival of 22 recurrent pterygium patients in high‐ (n = 13) and low‐risk (n = 9) groups based on FOXD2‐AS1 expression levels. F, OR for MVD and advanced stage, and HR for recurrence in pterygium patients based on FOXD2‐AS1 expression levels. The expression of FOXD2‐AS1 was quantified using qRT‐PCR. *P < 0.05, **P < 0.01. MVD, Microvessel density; ROC, Receiver operating characteristic; OR, odds ratio; HR, Hazard Ratio

qRT‐PCR analysis demonstrated that the expression of FOXD2‐AS1 was significantly higher in pterygium tissues than in adjacent conjunctiva tissues (*P* < 0.01, Figure 1A). Further, we found that patients at advanced stages tended to have higher expression of FOXD2‐AS1 (*P* < 0.05, Figure 1B) and increased microvessel density (*P* < 0.05, Figure 1C). FOXD2‐AS1 was not related to either the age or the gender of pterygium patients. The odds ratio (OR) of FOXD2‐AS1 for MVD and advanced stage is shown in Figure 1F.

### 3.2 Clinical value of FOXD2‐AS1 in predicting pterygium recurrence

First, the expression of FOXD2‐AS1 was significantly up‐regulated in recurrent pterygium patients compared to primary pterygium patients (*P* < 0.05, Figure 1A). Thus, we decided to assess whether FOXD2‐AS1 could be used as a predictive tool for recurrent pterygium, for which we used receiver operating characteristic (ROC) curves. The results demonstrated that FOXD2‐AS1 is effective in predicting pterygium recurrence. The best cut‐off level of FOXD2‐AS1 was 4.25, with an AUC of 0.702, sensitivity of 63.6% and specificity of 77.9% (*P* = 0.003, Figure 1D).

To evaluate the prognostic value of FOXD2‐AS1, recurrent pterygium patients were separated into ‘high‐risk’ (n = 13) and ‘low‐risk’ (n = 9) groups according to the best cut‐off level of FOXD2‐AS1. Kaplan‐Meier survival curves showed that patients in the high‐risk group had shorter recurrent‐free survival (*P* = 0.0014, Figure 1E). In addition, Cox regression analyses revealed a Hazard Ratio (HR) for recurrent FOXD2‐AS1 of 1.857 (*P* = 0.002, Figure 1F). Collectively, our results indicated that the FOXD2‐AS1 signature is a potential biomarker for the prediction of pterygium recurrence.

### 3.3 FOXD2‐AS1 induces proliferation of HPF‐R cells

**Figure 2:**
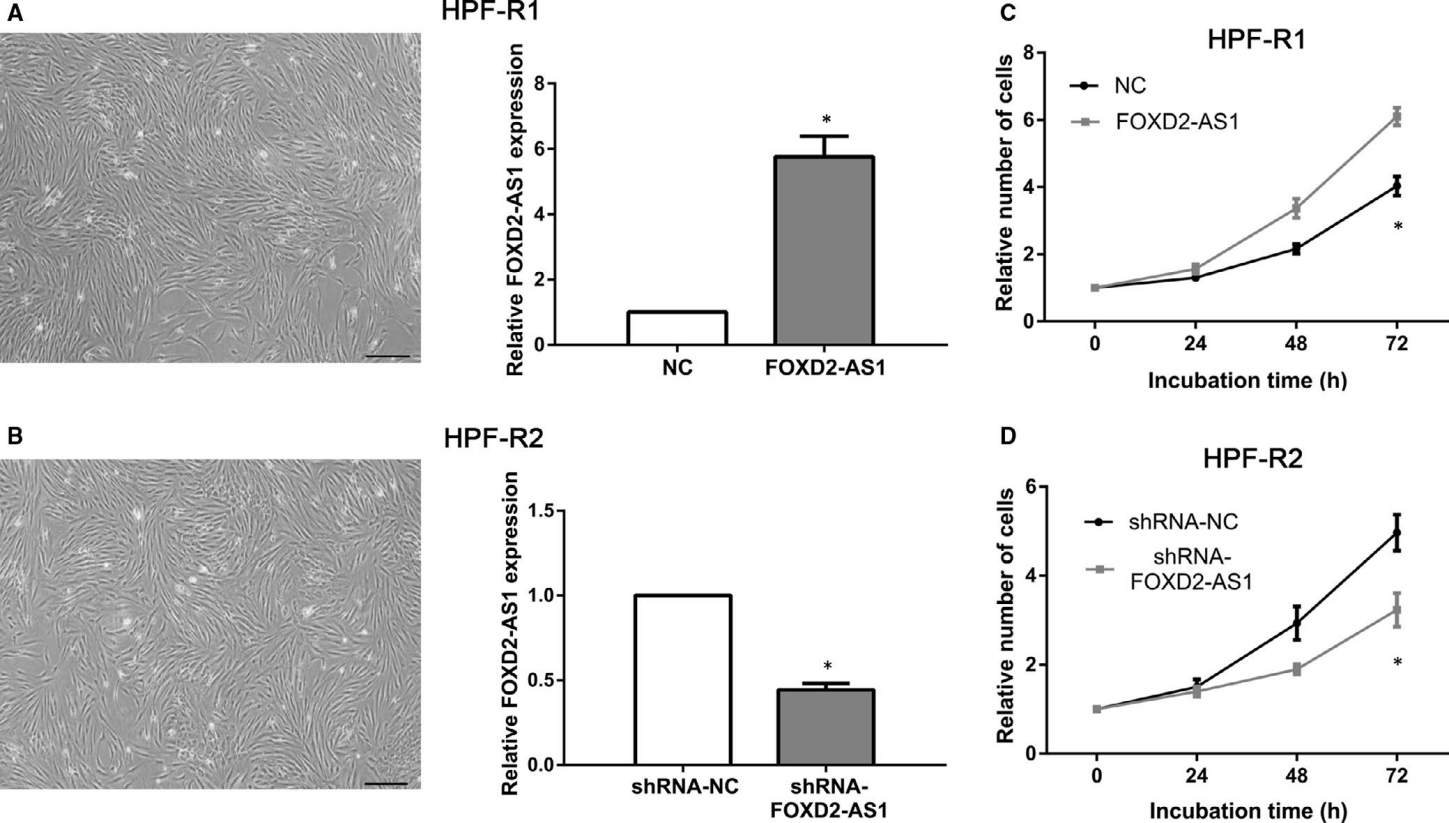
Effect of FOXD2‐AS1 overexpression on cell proliferation. A, B, Establishment of recurrent human pterygium cells with stable high expression of FOXD2‐AS1 (HPF‐R1) or stable low expression of FOXD2‐AS1 (HPF‐R2). The expression of FOXD2‐AS1 was quantified using qRT‐PCR. C, D, CCK‐8 assay was used to assess cell proliferation at 0, 24, 48 and 72 h. Scale bar, 100 µm. *P < 0.05

We isolated primary cells from recurrent pterygia and established cell lines (HPF‐R) with stable high or low expression of FOXD2‐AS1. As shown in Figure 2A, transfection with FOXD2‐AS1 plasmids resulted in a statistically significant overexpression of FOXD2‐AS1 in HPF‐R1 cells (*P* < 0.05). We also found that FOXD2‐AS1 expression was significantly decreased in transfected HPF‐R2 cells using shRNA‐FOXD2‐AS1 (*P* < 0.05, Figure 2B). Moreover, CCK‐8 assay revealed that HPF‐R1 cells overexpressing FOXD2‐AS1 had an increased proliferative ability compared to control cells (*P* < 0.05, Figure 2C), while transfection with shRNA‐FOXD2‐AS1 significantly repressed cell proliferation (*P* < 0.05, Figure 2D).

### 3.4 FOXD2‐AS1 suppresses apoptosis and promotes neoplasm growth in HPF‐R cells

**Figure 3:**
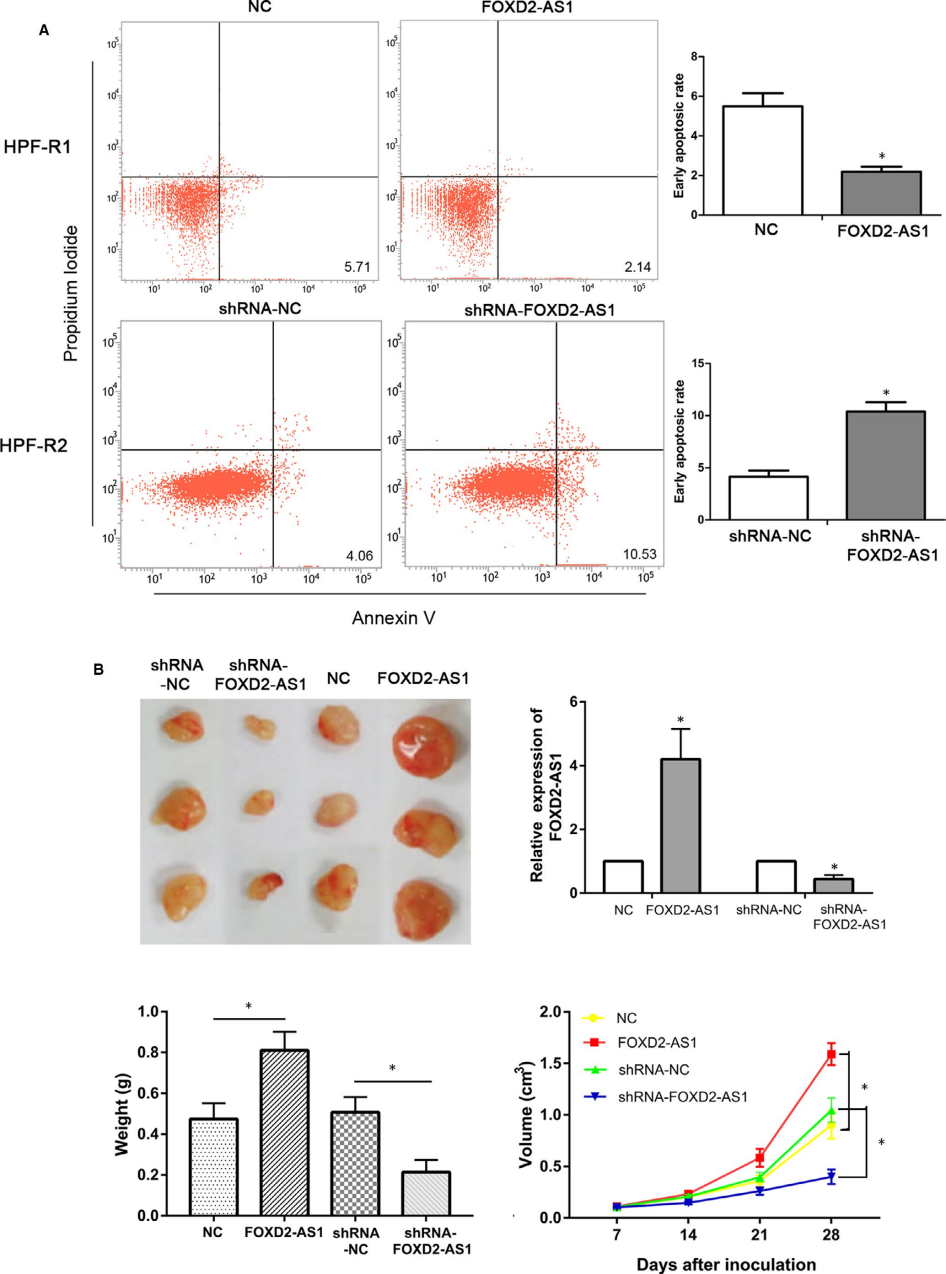
Effect of FOXD2‐AS1 on cell apoptosis and formation of new object. A, Flow cytometry showing the effect of FOXD2‐AS1 on the apoptosis of HPF‐R cells. B, Morphology of mice neoplasms dissected at day 28 after injection; FOXD2‐AS1 in neoplasms; neoplasm weight at day 29; neoplasm growth curves from day 7 to 28 after subcutaneous injection, with neoplasm volume calculated as V = (length × width^2^)/2. *P < 0.05

Flow cytometry analysis indicated that the early apoptosis rate of HPF‐R1 cells, which overexpress FOXD2‐AS1, was significantly decreased compared to that of control cells. In contrast, the early apoptosis rate of HPF‐R2 cells was significantly increased after FOXD2‐AS1 knockdown (*P* < 0.05, Figure 3A). Furthermore, the FOXD2‐AS1‐overexpressing xenografts progressed much faster and grew much larger than the controls in vivo. Contrarily, FOXD2‐AS1‐knockdown xenografts grew much smaller and slower than the controls in vivo (*P* < 0.05, Figure 3B).

### 3.5 FOXD2‐AS1 overexpression results in increased VEGF‐A expression in HPF‐R cells

**Figure 4:**
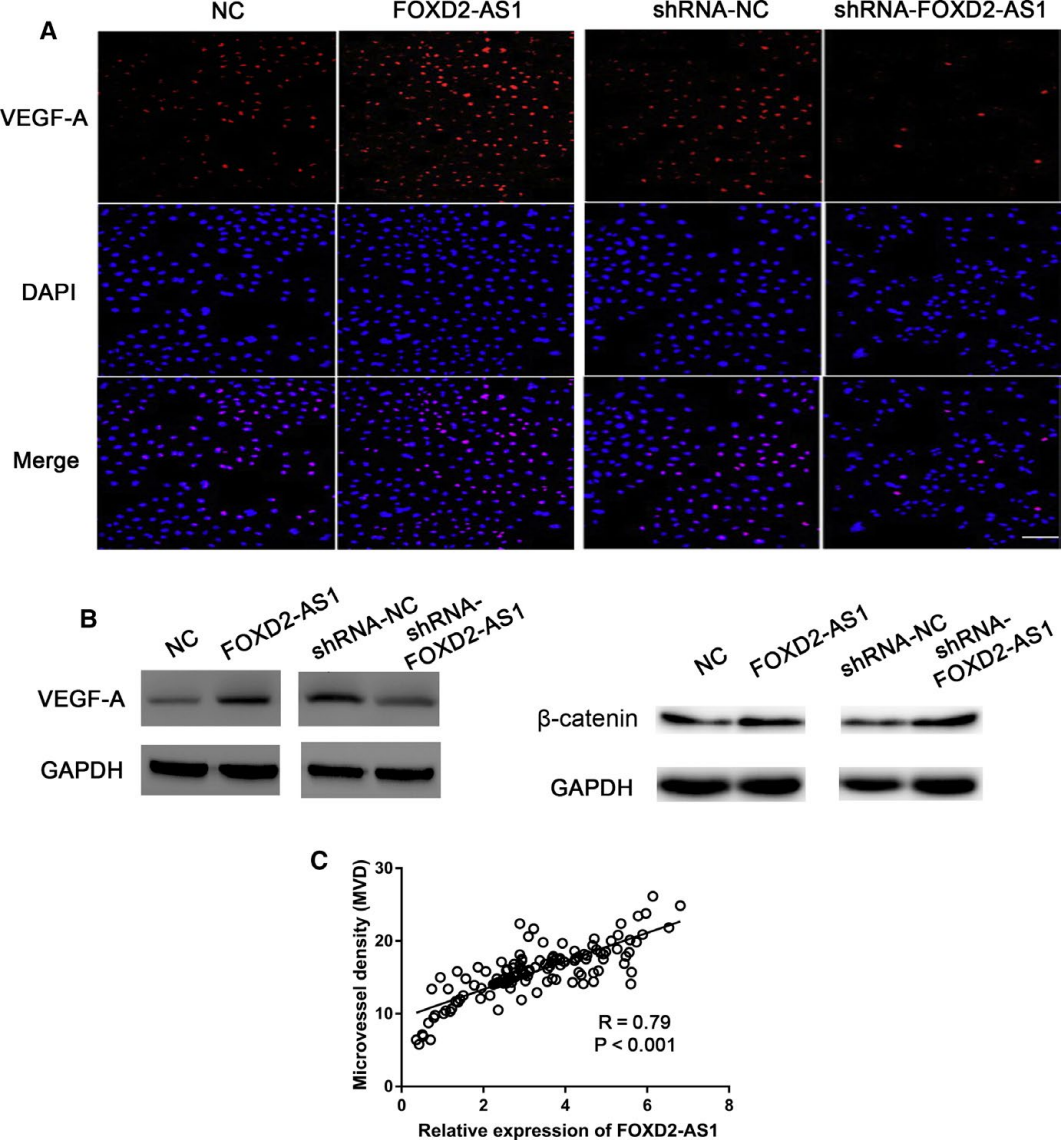
FOXD2‐AS1 regulates the expression of VEGF‐A. A, B, Immunofluorescence staining and Western blot analyses of FOXD2‐AS1 effect on the expression of VEGF‐A and β‐catenin. C, Correlation between FOXD2‐AS1 and MVD using Pearson's correlation assay in pterygium tissues (n = 126). Scale bar, 50 µm. *P < 0.05

Fluorescence immunoassays revealed an increase in VEGF‐A expression in HPF‐R1 cells (FOXD2‐AS1 overexpressing cells), whereas HPF‐R2 cells (FOXD2‐AS1 knockdown cells) showed reduced VEGF‐A expression (Figure 4A). Western blotting showed that the expression of the VEGF‐A protein was increased in HPF‐R1 cells and was suppressed in HPF‐R2 cells (Figure 4B). In addition, β‐catenin was up‐regulated in HPF‐R1 cells and down‐regulated in HPF‐R2 cells (Figure 4B). Moreover, Pearson's correlation analysis showed that FOXD2‐AS1 levels were positively correlated with MVD levels in pterygium patients (*R* = 0.79, *P* < 0.001, Figure 4C).

### 3.6 FOXD2‐AS1 targets miR‐205‐5p directly

**Figure 5:**
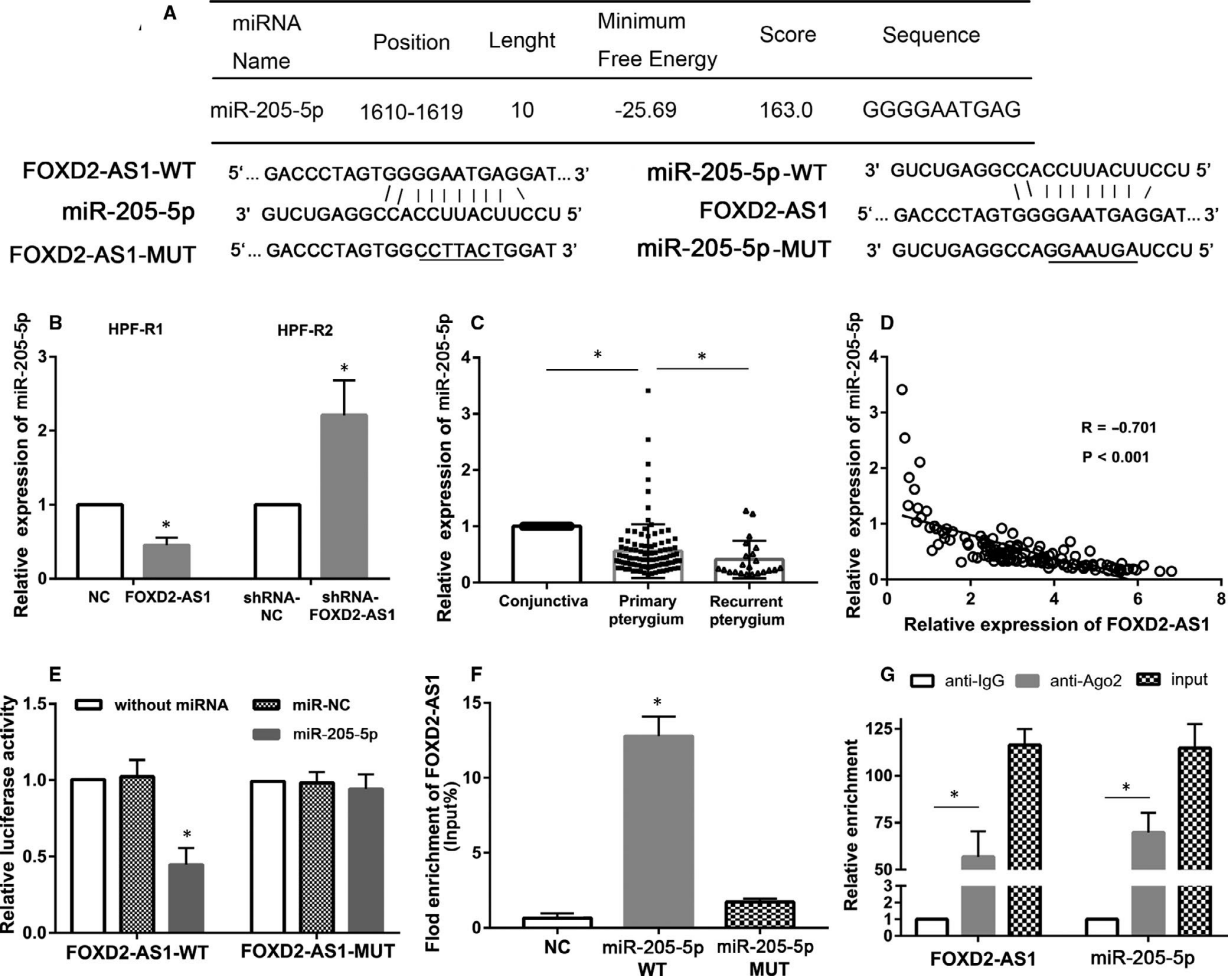
FOXD2‐AS1 functions as a molecular sponge for miR‐205‐5p in HPF‐R cells. A, Complementary sequence between FOXD2‐AS1 and miR‐205‐5p. B, The expression level of miR‐205‐5p in HPF‐R1 cells (overexpressing FOXD2‐AS1) and HPF‐R2 cells (FOXD2‐AS1 knockdown). C, The expression of miR‐205‐5p in adjacent conjunctiva tissues (n = 126), primary pterygium tissues (n = 104) and recurrent pterygium tissues (n = 22) using qRT‐PCR. D, Correlation analysis of FOXD2‐AS1 and miR‐205‐5p in pterygium tissues (n = 126). E, Luciferase activity in HPF‐R cells co‐transfected with miR‐205‐5p and FOXD2‐AS1‐WT, or with miR‐205‐5p and FOXD2‐AS1‐MUT. F, RNA pull‐down assays indicating the physical interaction between FOXD2‐AS1 and miR‐205‐5p. WT, wild type; MUT, Mutant type. G, RIP assays performed in HPF‐R cells. The relative RNA level in immunoprecipitates is presented as the fold change in Ago2 relative to IgG immunoprecipitates. *P < 0.05

First, we used bioinformatics tools to identify the target miRNAs of FOXD2‐AS1 and discovered a complementary sequence between FOXD2‐AS1 and miR‐205‐5p (Figure 5A). Next, we confirmed that miR‐205‐5p expression was significantly down‐regulated in HPF‐R1 cells and significantly increased in HPF‐R2 cells compared with their control groups, respectively (*P* < 0.05, Figure 5B). We also used pterygium tissues and adjacent conjunctiva tissues to perform qRT‐PCR assays, which revealed that miR‐205‐5p was not only lower in primary pterygium tissues than in adjacent conjunctiva tissues, but also lower in recurrent pterygium tissues than in primary pterygium tissues (*P* < 0.05, Figure 5C). Moreover, Pearson's correlation analysis showed that the levels of miR‐205‐5p and FOXD2‐AS1 were negatively correlated (*R* = −0.701, *P* < 0.001, Figure 5D).

Furthermore, Dual‐luciferase reporter assay showed that the luciferase activities were significantly decreased in the FOXD2‐AS1‐WT plasmid and miR‐205‐5p mimics co‐transfected group compared with FOXD2‐AS1‐MUT plasmid and miR‐205‐5p mimics co‐transfected group (*P* < 0.05, Figure 5E). Moreover, the results from the RNA pull‐down assays showed that the biotin‐labelled miR‐205‐5p‐WT mimics pulled down more FOXD2‐AS1‐WT than the biotin‐labelled miR‐205‐5p‐MUT mimics (*P* < 0.05, Figure 5F). Additionally, RIP experiments showed enrichment of miR‐205‐5p and FOXD2‐AS1 in immunoprecipitated Ago2, which is a key protein with ceRNA function, compared with the control IgG (*P* < 0.05, Figure 5G).

### 3.7 VEGF‐A is a direct target of miR‐205‐5p

**Figure 6:**
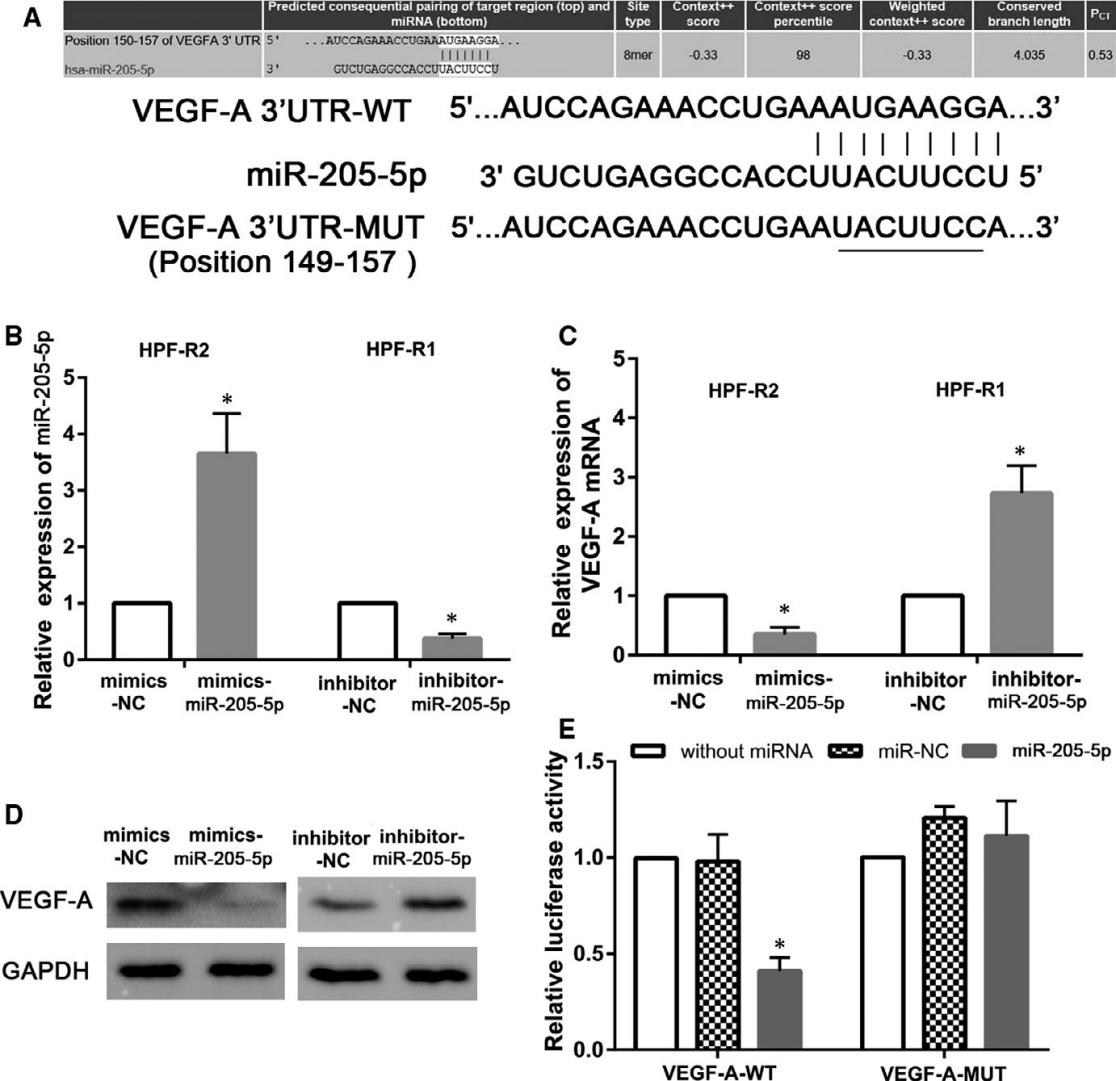
VEGF‐A is directly regulated by miR‐205‐5p. A, Putative binding sites of miR‐205‐5p in VEGF‐A 3‐’UTR. B, Expression levels of miR‐205‐5p after transfection of mimics‐miR‐205‐5p in HPF‐R2 cells, and inhibitor‐miR‐205‐5p in HPF‐R1 cells. C, D qRT‐PCR analysis of VEGF‐A mRNA expression level and western blot analysis of VEGF‐A protein expression level in the HPF‐R cell lines transfected with mimics‐miR‐205‐5p or inhibitor‐miR‐205‐5p, respectively. E, Relative luciferase activity measured in HPF‐R cells after co‐transfection of the VEGF‐A luciferase construct (WT or MUT) with either miR‐205‐5p or control. *P < 0.05

First, we predicted that VEGF‐A could be a downstream target of miR‐205‐5p using bioinformatics tools (Figure 6A). Next, we examined the effects of miR‐205‐5p mimics and inhibitor. RT‐PCR results showed that the expression of miR‐205‐5p in the miR‐205‐5p mimics group and miR‐205‐5p inhibitor group was 3.8‐fold and 0.54‐fold that of their control groups, respectively (*P* < 0.05, Figure 6B).

Furthermore, qRT‐PCR and Western blotting showed a significant reduction in the mRNA and protein levels of VEGF‐A in HPF‐R cells transfected with the miR‐205‐5p mimics compared to control cells (*P* < 0.05, Figure 6C). In contrast, the mRNA and protein levels of VEGF‐A were dramatically up‐regulated in HPF‐R cells in which miR‐205‐5p was inhibited, compared with the control cells (*P* < 0.05, Figure 6D). Indeed, luciferase reporter assays with WT and MUT type VEGF‐A 3’‐UTR binding sites for miR‐205‐5p also showed decreased luciferase activity with WT VEGF‐A 3’‐UTR upon miR‐205‐5p overexpression, while MUT VEGF‐A 3’‐UTR binding sites had no effect on luciferase activity (*P* < 0.05, Figure 6E).

### 3.8 Effect of FOXD2‐AS1 and miR‐205‐5p on cell proliferation

**Figure 7:**
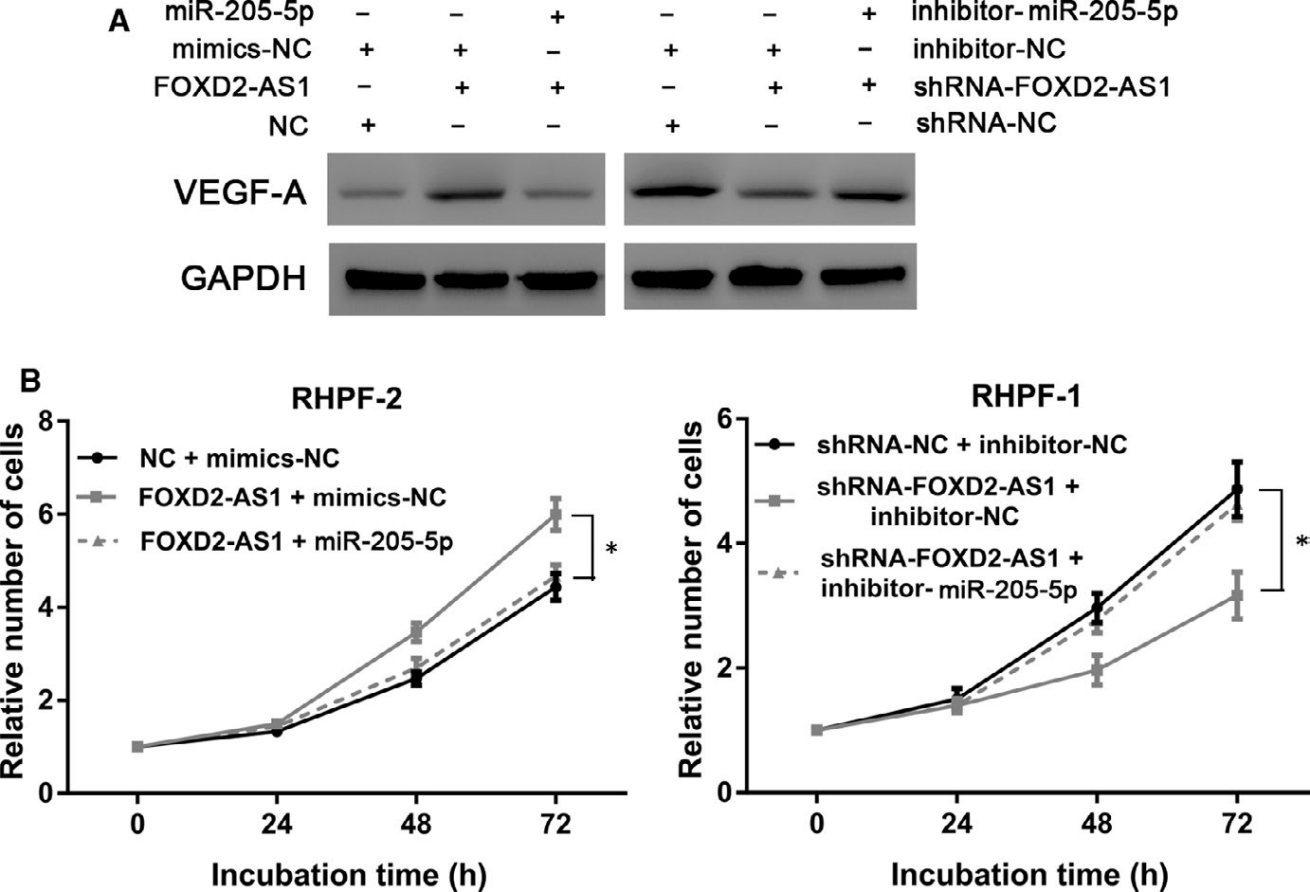
The proliferation‐promoting function of FOXD2‐AS1 is dependent on miR‐205‐5p and VEGF‐A. A, To reverse the regulatory effect of FOXD2‐AS1 on miR‐205‐5p, mimics‐miR‐205‐5p or inhibitor‐miR‐205‐5p were transfected into HPF‐R1 cells (overexpressing FOXD2‐AS1) and HPF‐R2 cells (FOXD2‐AS1 knockdown). Western blot showing the expression levels of VEGF‐A after the regulatory effect of FOXD2‐AS1 on miR‐205‐5p was attenuated. B, CCK‐8 assay showing HPF‐R cell proliferation after the regulatory effect of FOXD2‐AS1 on miR‐205‐5p was attenuated. *P < 0.05

We examined the effect of FOXD2‐AS1/mimics‐miR‐205‐5p and shRNA‐FOXD2‐AS1/inhibitor‐miR‐205‐5p, co‐transfections on VEGF‐A level and cell proliferation. The data demonstrated that co‐transfection with FOXD2‐AS1 and mimics‐miR‐205‐5p significantly reduced VEGF‐A levels and cell proliferation compared with transfection with FOXD2‐AS1 alone. Not surprisingly, co‐transfection with shRNA‐FOXD2‐AS1 and inhibitor‐miR‐205‐5p significantly increased VEGF‐A levels and cell proliferation compared with shRNA‐FOXD2‐AS1 transfection alone (*P* < 0.05, Figure 7).

### 3.9 H3K27 acetylation activates FOXD2‐AS1 expression

**Figure 8:**
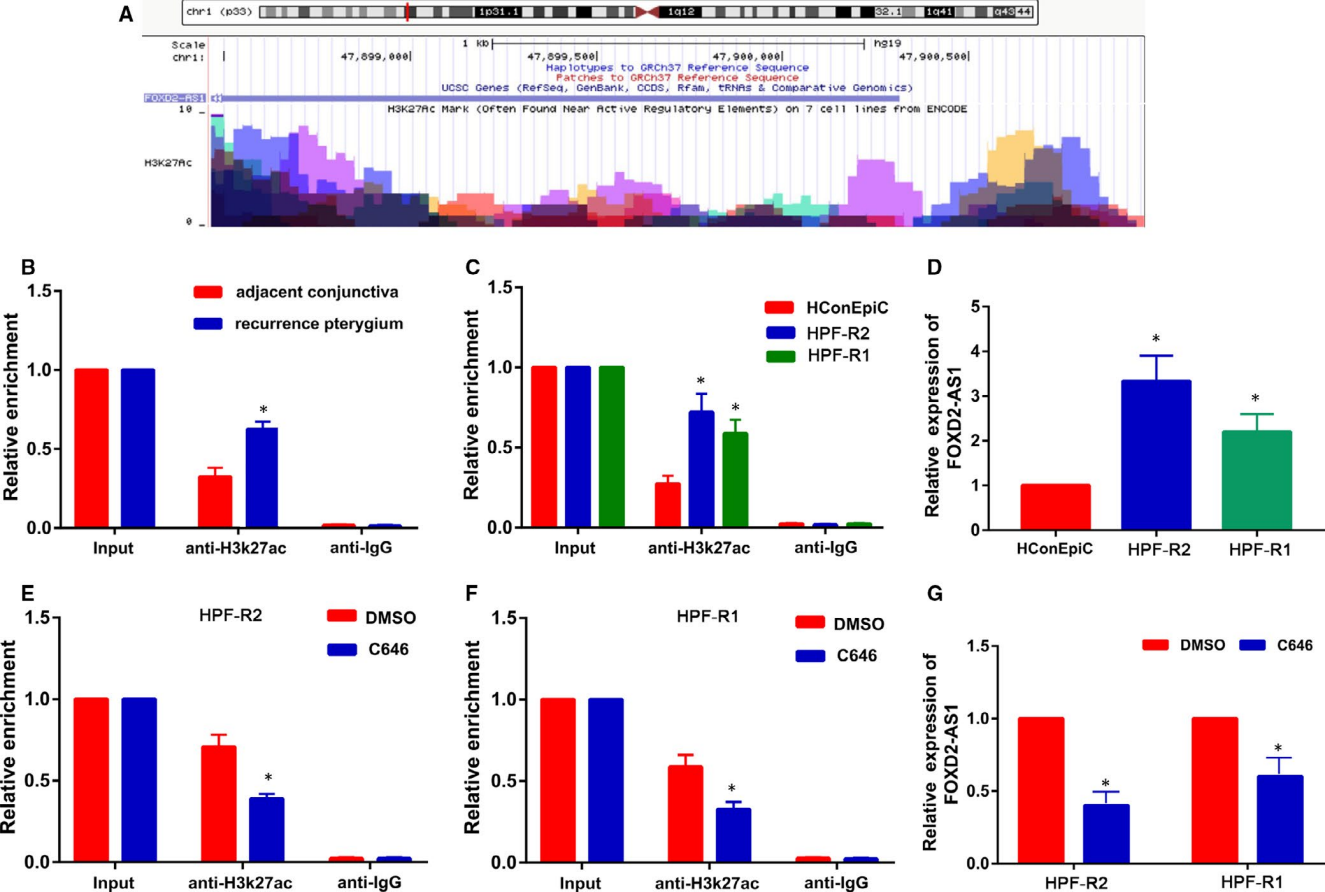
FOXD2‐AS1 is activated by H3K27 acetylation at the promoter region. A, Bioinformatics analysis showing high enrichment of H3K27ac at the FOXD2‐AS1 promoter. B, C, ChIP assays detecting H3K27 acetylation at the FOXD2‐AS1 promoter in pterygium tissues and cells. D, Expression of FOXD2‐AS1 in conjunctival cell line (HconEpiC) and pterygium cell lines (HPF‐R) using qRT‐PCR. E, F, ChIP assays detecting H3K27 acetylation at the FOXD2‐AS1 promoter after treatment of HPF‐R cells with C646 (histone acetyltransferase inhibitor). G, FOXD2‐AS1 expression in C646‐treated HPF‐R cells using qRT‐PCR. *P < 0.05

To further understand the mechanism behind the high FOXD2‐AS1 levels in HPF‐R cells, we performed a bioinformatics analysis (http://genome.ucsc.edu/) and found a high enrichment of H3K27ac in the FOXD2‐AS1 promoter (Figure 8A). Further, we conducted a ChIP assay using 22 paired tissues and found a significantly increased level of H3K27ac at the FOXD2‐AS1 promoter in recurrent pterygium tissues compared with adjacent conjunctiva tissues (*P* < 0.05, Figure 8B). Furthermore, the H3K27ac enrichment was also significantly increased in HPF‐R cells compared with HconEpiC cells (conjunctival cell line) (*P* < 0.05, Figure 8C). As expected, FOXD2‐AS1 expression levels were significantly up‐regulated in HPF‐R cells compared with HconEpiC cells (*P* < 0.05, Figure 8D). Moreover, the enrichment in H3K27ac was dramatically lower in C646‐treated HPF‐R cells compared with the control cells (*P* < 0.05, Figure 8E,F). Furthermore, C646‐treated HPF‐R cells showed a decreased expression of FOXD2‐AS1 compared with control cells (*P* < 0.05, Figure 8G).

## 4 DISCUSSION

Aberrant expression of FOXD2‐AS1 is involved in cancer initiation, progression and metastasis.^10^, ^11^ Pterygium cells are tumour‐like transformed cells that compared to normal fibroblasts, grow much more rapidly in medium without high concentrations of serum and can grow in a semisolid agar.^15^ However, the role of FOXD2‐AS1 in pterygium progression and prognosis has not been clarified. In this study, we observed up‐regulation of FOXD2‐AS1 expression in pterygium tissues. Further, we found that the FOXD2‐AS1 level was positively correlated with advanced stages and increased MVD in pterygium tissues. Logistic regression analysis demonstrated that FOXD2‐AS1 is a risk factor for advanced stages and increased MVD. Indeed, an inflamed stage and increased MVD are crucial factors for recurrent pterygium. Further, we found that FOXD2‐AS1, which is related to the inflamed stage and increased MVD, is associated with poor recurrent‐free survival. Collectively, our data demonstrate that FOXD2‐AS1 may be involved in pterygium progression and recurrence.

Growing evidence suggests that lncRNAs such as FOXD2‐AS1 could serve as potential biomarkers with high sensitivity and specificity for disease detection and diagnosis.^9^, ^10^, ^16^ Based on this, we explored the potential application of FOXD2‐AS1 as a biomarker for the prediction of recurrent pterygium. First, we found that FOXD2‐AS1 expression levels were significantly higher in recurrent pterygium tissues than primary pterygium tissues. Next, Kaplan‐Meier analysis confirmed that patients with high FOXD2‐AS1 expression had significantly shorter recurrent‐free survival than those with low expression. Cox regression analyses also proved that FOXD2‐AS1 was a risk factor for recurrence. Last, the specificity of FOXD2‐AS1 in predicting pterygium recurrence was confirmed in the ROC curve. Thus, we suggest that FOXD2‐AS1 may be an ideal biomarker for recurrent pterygium.

Then, we decided to explore the function of FOXD2‐AS1 in HPF‐R cells by conducting gain‐ and loss‐of‐function experiments in vitro and in vivo. Our data indicated that FOXD2‐AS1 might promote proliferation and suppress apoptosis of HPF‐R cells, which is consistent with the results from previous studies on FOXD2‐AS1 in cancer.^10^, ^11^

Lei^17^ showed that FOXD2‐AS1 can promote the proliferation and migration of hepatocellular carcinoma by activating the Wnt/β‐catenin signalling pathway. Rong^18^ found that FOXD2‐AS1 can promote non‐small cell lung cancer cell growth via regulating Wnt/β‐catenin signalling. In this study, we also showed that FOXD2‐AS1 can regulate β‐catenin in HPF‐R cells, indicating that the Wnt/β‐catenin pathway may be involved in the regulation of HPF‐R cell function by FOXD2‐AS1.

The pathogenesis of pterygium is an active process associated with cellular proliferation and angiogenesis. The evidence indicates that VEGF is increased in pterygium and may contribute to its progression and recurrence by increasing angiogenesis and growth. For instance, Wu^19^ reported that VEGF can activate fibroblasts in pterygium by overexpressing low‐density lipoprotein receptors. Furthermore, a systematic review and network meta‐analysis of 2483 patients showed that bevacizumab, a vascular growth factor antagonist, could effectively prevent recurrence following pterygium excision.^20^ Given these studies and our results on cell function, we wondered whether FOXD2‐AS1 could regulate the expression of VEGF‐A. First, we found that FOXD2‐AS1 could significantly inhibit VEGF‐A expression in HPF‐R cells. Furthermore, we found that FOXD2‐AS1 expression positively correlated with MVD expression in pterygium tissues. Since it has previously been reported that VEGF‐A is highly expressed in pterygium tissues with high MVD,^21^ we suggest that FOXD2‐AS1 up‐regulation contributes to HPF‐R cell growth at least in part by regulating VEGF‐A expression.

Competing endogenous RNAs (ceRNAs) are stable lncRNAs that accumulate in large numbers and control post‐transcriptional gene expression in different ways, including acting as decoys or sponges for miRNAs.^22^, ^23^ Previous studies have demonstrated that FOXD2‐AS1 is associated with cell proliferation and apoptosis by acting as ceRNAs. For example, FOXD2‐AS1 acts as a sponge of miR‐185‐5p to regulate HMGA2 and influence the PI3K/Akt signalling pathway in glioma.^24^ FOXD2‐AS1 can also promote the growth of chondrocytes by targeting the miR‐206/CCND1 axis.^25^ More importantly, a study based on three GEO data sets indicated the construction of a ceRNA network in pterygium development, although its characteristics remained rather unclear.^26^

In this study, we found that FOXD2‐AS1 could be enriched by Ago2 in HPF‐R cells, indicating that FOXD2‐AS1 might be involved in the ceRNA network. Thus, we speculated that FOXD2‐AS1 may also function as a ceRNA of microRNAs in the regulation of VEGF‐A expression and the proliferation of HPF‐R cells. To test this hypothesis, we used online bioinformatics databases to predict combinations of sequences and found that the FOXD2‐AS1 sequence contains potential binding sites for miR‐205‐5p. Interestingly, bioinformatics prediction tools also indicated that VEGF‐A might be a downstream target of miR‐205‐5p.

Then, we first demonstrated the regulatory effect of FOXD2‐AS1 on miR‐205‐5p. We proved that miR‐205‐5p is significantly down‐regulated in pterygium tissues, especially in recurrent pterygium tissues. The expression of FOXD2‐AS1 and miR‐205‐5p was negatively correlated in pterygium tissues. Furthermore, FOXD2‐AS1 had an inhibitory effect on miR‐205‐5p expression in HPF‐R cells. Importantly, luciferase reporter and RNA pull‐down assays confirmed that miR‐205‐5p is a target of FOXD2‐AS1 in HPF‐R cells. Thus, FOXD2‐AS1 may contribute to the down‐regulation of miR‐205‐5p by direct bonding.

Second, we revealed the regulatory effect of miR‐205‐5p on VEGF‐A. We found that miR‐205‐5p reduces both mRNA and protein VEGF‐A levels in HPF‐R cells. In addition, the results from the luciferase reporter assay also revealed that VEGF‐A may be one of the direct targets of miR‐205‐5p in HPF‐R cells.

Furthermore, we co‐transfected FOXD2‐AS1 and miR‐205‐5p in HPF‐R cells and found that miR‐205‐5p could reverse the cell growth promoting effects of FOXD2‐AS1 in HPF‐R cells through restoring the inhibitory effect of miR‐205‐5p on VEGF‐A expression. Overall, these data indicated that an interaction with miR‐205‐5p is necessary for FOXD2‐AS1 to inhibit cell growth and promote apoptosis via the VEGF‐A pathway in HPF‐R cells.

In summarize, FOXD2‐AS1 regulated miR‐205‐5p by acting as ceRNAs. MiR‐205‐5p inhibited VEGF‐A protein synthesis by binding to the 3' untranslated region (3'UTRs) of the mRNA through partial complementary binding. FOXD2‐AS1 affected HPF‐R cell function by indirectly regulating VEGF‐A.

Last, we explored the mechanism behind the high FOXD2‐AS1 expression in recurrent pterygium. Recent studies have shown that the aberrant expression of lncRNAs is attributed to acetylation‐mediated transcriptional activation.^27^, ^28^, ^29^ Histone acetylation leads to the weakening of the DNA‐histone interaction and the subsequent activation of transcription.^30^ Therefore, we analysed the promoter region of FOXD2‐AS1 by bioinformatics analysis and identified that H3K27ac was highly enriched in this region. Furthermore, we confirmed that the enrichment level of H3K27ac at the FOXD2‐AS1 promoter was notably increased in both HPF‐R cells and recurrent pterygium tissues, resulting in FOXD2‐AS1 up‐regulation. The histone acetylation process is controlled by histone acetyltransferases and histone deacetylases. Therefore, we investigated whether the H3K27ac modification is involved in FOXD2‐AS1 expression using C646 (histone acetyltransferase inhibitor). As expected, C646 reduced the H3K27ac enrichment at the FOXD2‐AS1 promoter, and consequently, resulted in a decreased expression of FOXD2‐AS1. Overall, our results strongly support that the enrichment of H3K27ac at the FOXD2‐AS1 promoter region leads to the up‐regulation of FOXD2‐AS1 expression.

In summary, our study demonstrates for the first time that FOXD2‐AS1 is activated by H3K27 acetylation and that this activation leads to enhanced proliferation and suppression of apoptosis in cell lines established from recurrent pterygium fibroblasts. Moreover, we show that this action is exerted through the regulation of the miR‐205‐VEGF pathway. Based on our results, we propose FOXD2‐AS1 as a potential novel therapeutic target and diagnosis biomarker for recurrent pterygium.

## CONFLICT OF INTEREST

No potential conflicts of interest were disclosed.

## AUTHOR CONTRIBUTION

**Yali Gao:** Conceptualization (supporting); Formal analysis (supporting); Funding acquisition (lead); Investigation (lead); Methodology (lead); Project administration (supporting). **Xiaoling Luo:** Formal analysis (supporting). **Jun Zhang:** Conceptualization (lead); Formal analysis (lead); Funding acquisition (supporting); Investigation (supporting); Methodology (supporting); Project administration (lead).

## Supporting information

Table S1

Table S2

## Data Availability

The data used to support the findings of this study are available from the corresponding author upon request.
