# Supplementary material for: Activation of LncRNA FOXD2‐AS1 by H3K27 acetylation regulates VEGF‐A expression by sponging miR‐205‐5p in recurrent pterygium
Source: J Cell Mol Med. 2020 Oct 23;24(24):14139–51. doi: 10.1111/jcmm.16024 (PMC7754060; doi:10.1111/jcmm.16024)
Supplement: Supplementary file 1 — Table S1 [file JCMM-24-14139-s001.doc]

Table S1 Oligonucleotide sequence

| Name | Sequence |
| --- | --- |
| FOXD2-AS1 shRNA | 5'-GATCCGGGATCTAATTAAGTCAATCTTCAAGAGAGATTGACTTAATTAGATCCCTTTTTTG-3' (sense)  5'-AATTCAAAAAAGGGATCTAATTAAGTCAATCTCTCTTGAAGATTGACTTAATTAGATCCCG-3' (antisense) |
| NC shRNA | 5'-GATCCGTTCTCCGAACGTGTCACGTTTCAAGAGAACGTGACACGTTCGGAGAACTTTTTTG-3' (sense)  5'-AATTCAAAAAAGTTCTCCGAACGTGTCACGTTCTCTTGAAACGTGACACGTTCGGAGAACG-3' (antisense) |
