# Supplementary material for: Activation of LncRNA FOXD2‐AS1 by H3K27 acetylation regulates VEGF‐A expression by sponging miR‐205‐5p in recurrent pterygium
Source: J Cell Mol Med. 2020 Oct 23;24(24):14139–51. doi: 10.1111/jcmm.16024 (PMC7754060; doi:10.1111/jcmm.16024)
Supplement: Supplementary file 2 — Table S2 [file JCMM-24-14139-s002.doc]

Table S2 PCR primers

| Gene Name | Forward Primer (5’-3’) | Reverse Primer(5’-3’) |
| --- | --- | --- |
| FOXD2-AS1 | 5′-TGGACCTAGCTGCAGCTCCA-3′ | 5′-AGTTGAAGGTGCACACACTG-3′ |
| GAPDH | 5′-TGCACCACCAACTGCTTAGC-3′ | 5′-GGCATGCACTGTGGTCATGAG-3′ |
| FOXD2-AS1  For ChIP | 5′-GAAGTGAGTACGTGAGCGGC-3′ | 5′-TCGTGATTCGGGATGCACAG-3′ |
